# Human Factor Errors in the use of the PAWPER Tape Systems: An Analysis of Inter-Rater Reliability

**DOI:** 10.7759/cureus.12468

**Published:** 2021-01-04

**Authors:** Mike Wells, Lara N Goldstein

**Affiliations:** 1 Emergency Medicine, University of the Witwatersrand, Johannesburg, ZAF

**Keywords:** pawper xl tape, interrater reliability

## Abstract

Background

International guidelines have promoted the use of length-based tapes for emergency weight estimation in children. This is primarily because of a perception that more modern methods might require more training than can practically be achieved. This study aimed to evaluate the inter-rater reliability of novice users of the PAWPER XL (paediatric advanced weight-prediction in the emergency room) tape as an indicator of the ability to use the device effectively with limited training.

Methods

This was a secondary analysis of unpublished data from three previous studies. Inter-rater reliability analyses were performed for each study for the accuracy of weight estimations and for the assignment of body habitus score. Inter-rater reliability was analysed using percentage agreement and Cohen’s Kappa for Study 1 and intraclass correlation (ICC) for Study 2 and Study 3. A value of <0.7 was considered to indicate inadequate agreement, 0.7 to 0.89 was considered to indicate good agreement and ≥0.9 was considered to indicate excellent agreement.

Main Results

A total of 7034 data points were available for analysis in the three studies. In Study 1, the percentage agreement of an accurate weight estimation in 50 children, with two raters, was 47/50 (94%) with a Cohen’s Kappa of 0.93 (0.81 - 1.0). In Study 2, the ICC for 6720 habitus score assessments, with 112 raters, was 0.73 (0.68 - 0.80). In Study 3, the ICC for 264 weight estimations (in terms of an accurate weight estimation, with 33 raters) was 0.88 (0.72 - 0.97).

Conclusions

There was good inter-rater reliability in the assessment of habitus and the accuracy of weight estimation for the PAWPER XL tape in this secondary analysis. The findings suggest that reasonable proficiency with the system can be achieved with minimal training. It is therefore unlikely that systems such as this require too much training to be practical in emergency care.

## Introduction

Almost all drug doses in children are based on their body weight. When drugs need to be administered in an emergency, it is often impossible to weigh a child, and therefore an estimation of weight is required to permit drug dose calculations [1]. The oldest methods of paediatric weight estimation are age-based formulas, whose proponents claim two theoretical benefits. Firstly, they are simple to use. Secondly, they allow for prehospital personnel to forewarn Emergency Department doctors of the age of a child they are bringing in, so that drug doses can be precalculated and prepared [2]. The current evidence, however, does not support this thinking. Recollection errors and calculation errors are common when age-formulas are used during emergencies as their perceived simplicity is not borne out by empirical evidence [3]. More importantly, age formulas do not provide an accurate estimate of children’s weight and, as a result, resultant drug doses will be inaccurate [4]. Age-based weight estimation formulas should not be used, when newer and better methods are available [5].

There is now abundant evidence that two-dimensional length- and habitus-based methods of paediatric weight estimation are superior to age-based or length-based methods [6]. The most well-researched of the length- and habitus-based methods are the PAWPER XL (paediatric advanced weight-prediction in the emergency room) tape and the Mercy method [7, 8]. However, while the accuracy of weight estimation is clearly important, the usability of a weight estimation system is also important [9]. A weight estimation system that is difficult to use, or one that requires a great deal of training, would be less useful than one that is easy to use with minimal training. Furthermore, a system that produces sizeable variations in accuracy between users would be less useful than one with more consistently reliable results [10, 11]. However, the complexity of a weight estimation system is not necessarily the primary determinant of its usability. Somewhat surprisingly, there is compelling evidence that even simple length-based weight estimation systems, such as the Broselow tape, are vulnerable to user-errors (human factor errors) which could ultimately harm the patient due to medication dosing errors [12, 13]. A recent systematic review and meta-analysis on the Broselow tape highlighted its inaccuracy relative to the dual length- and habitus-based weight estimation methods. It also confirmed the detrimental impact of user-errors on the performance of the tape when used by untrained individuals [14].

The Mercy method, a very accurate weight estimation method that makes use of measurements of humeral length and mid-arm circumference, has shown significant differences in accuracy between non-expert users [15]. The PAWPER XL tape makes use of recumbent body length and a reference-image assisted assessment of body habitus to estimate total body weight. Therefore, there is the potential for either the measurement of length and/or the assessment of habitus to differ between users, which could affect the accuracy of the system. However, there is only a single study on the usability and human factor errors related to the use of the PAWPER XL tape [16]. This preliminary work showed reasonably consistent accuracy with the use of the PAWPER XL system, but also that some users were more accurate than others.

There is evidence that the PAWPER XL tape is very accurate when operated by those well-trained and practised with its use [7, 17]. However, it is important to establish the human factor errors that could potentially be associated with its use by untrained or minimally trained individuals. Since an accurate drug dose depends on an accurate estimation of weight, this is a critical consideration [18].

The aim of this study was simple: to evaluate the inter-rater reliability of the PAWPER XL tape system when used by non-expert users. In addition, the study aimed to provide a comprehensive evaluation of each aspect of the methodology of the PAWPER XL tape. This could provide a perspective of the degree to which human factors influence the accurate use of the system.

## Materials and methods

**Figure 1:**
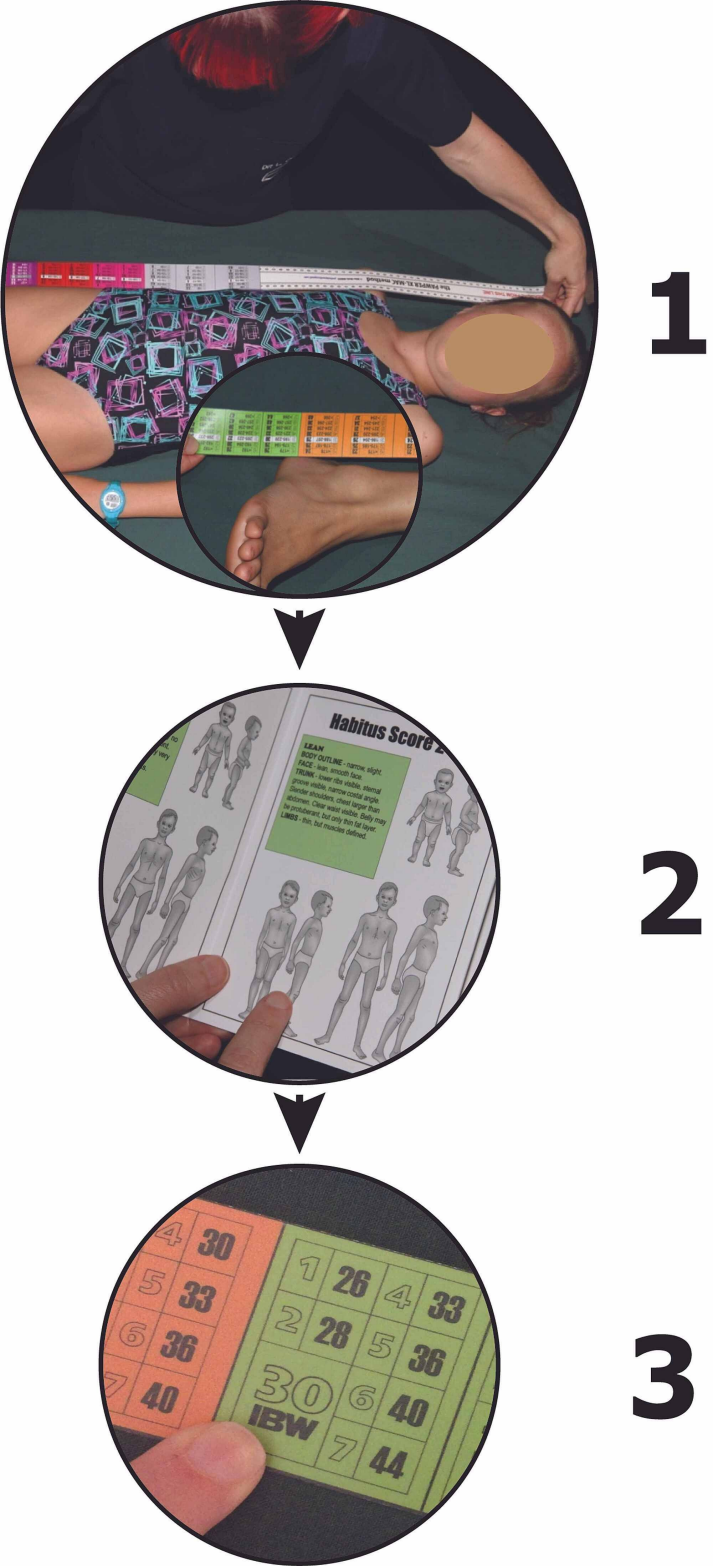
How the PAWPER XL tape is used. The figure shows the three steps involved in the use of the PAWPER XL tape. Step 1 - the child is measured with the tape from head to heel. Step 2 - the child's habitus is assessed using reference figural images. These reference images appear on the tape itself as well as on a card that accompanies the PAWPER XL tape. A habitus score (HS) is then assigned. HS1 is a very underweight child, HS3 is a child with ideal body weight, HS5 is an obese child and HS7 is a severely obese child. Step 3 - the child's estimated weight is read off the PAWPER XL tape at the length measured and for the habitus score assigned.

This was a secondary analysis of previously unpublished data from three studies in which either the original PAWPER tape or the newer PAWPER XL tape was evaluated when used by non-expert users [16, 19, 20]. Non-expert users were considered to be those who had never used the tape previously or those that had never had formal training in the use of the tape. Each study was used to evaluate a different aspect of the human factors influencing the performance of the tape. The primary outcomes for each study were used as the primary outcomes in the interrater reliability analysis. The details of how the PAWPER XL tape is used is shown in Figure 1. The original PAWPER tape is very similar to the newer PAWPER XL tape, but is shorter and has fewer habitus score categories. Both tapes are used in the same way.

Ethical approval for the secondary analysis was provided by the Human Research Ethics Committee of the University of the Witwatersrand (protocol M120486).

Study 1 

This study was conducted in an academic Emergency Department in Johannesburg, South Africa [20]. A convenience sample of 453 children aged from 0 to 13 years were recruited and had weight estimations with the original PAWPER tape. Weight estimations were performed by a total of 18 different doctors and nursing staff on duty in the Emergency Department. The majority of estimations were performed by three non-investigators. In order to determine inter-rater reliability, a random sample of 50 children had weight estimations performed twice, by different assessors. The percentage agreement method and Cohens kappa method were used to evaluate the inter-rater reliability. In addition, the difference in accuracy of weight estimations was determined between "major" data collectors and "minor" data collectors. Major data collectors were empirically determined to have been those who performed >50 estimations and minor data collectors those who had performed <30 estimations. The proportion of weight estimations accurate to within 10% and 20% of actual weight (defined as an accurate weight estimation) was compared between the groups as an indicator of reliability as determined by degree of practice and experience.

Study 2 

In this study, 112 volunteer doctors and paramedics were asked to assess the body habitus score of 60 children [19]. The group of doctors included junior emergency medicine medical officers and registrars as well as paediatric registrars. The participants used a simple gestalt assessment of habitus in 30 children and used reference images to assist assessment in the other 30 children. The habitus score is one of the components from which PAWPER XL tape weight estimations are generated in clinical practice. It is the most subjective part of the PAWPER XL tape methodology. A total of 6720 valid habitus score estimations were available for evaluation. The inter-rater reliability of the assessment of habitus score was determined using intraclass correlation (ICC). Since the PAWPER XL tape is generally used by a single individual in a clinical setting, a model with two-way random effects, absolute agreement and an individual rating, rather than an average rating, was used (ICC (2,1)) to generalise to the inter-rater reliability in a clinical setting [21]. While a model using and multiple raters (ICC (2,k)) can be used for this type of analysis, it is of limited value as the correlations are generally very high with large sample sizes.

Subgroup analyses were performed for subgroups of doctors, paediatricians and advanced life-support paramedics. 

Study 3 

This study included 33 participants (doctors and advanced life-support paramedics), each of whom participated in eight realistic simulations of paediatric emergencies in which weight was estimated using the PAWPER XL tape methodology [16]. The scenarios included children in different positions (supine, sitting, lateral recumbency) and with different degrees of cooperation (cooperative, passively uncooperative, actively uncooperative). A total of 264 weight estimations were then evaluated for inter-rater agreement in terms of the accuracy of weight estimations (proportions of weight estimations within 10% and 20% of actual weight) using intraclass correlation. Since the PAWPER tape is generally used by a single individual, a model with two-way random effects, absolute agreement and an individual rater, rather than an average rating, was also used (ICC (2,1)) [21].

Statistical analysis

Inter-rater reliability analyses were performed as described for each study above. For the Cohen’s kappa and the intraclass correlation, a value of <0.7 was considered to indicate inadequate agreement, 0.7 to 0.89 was considered to indicate good agreement and ≥0.9 was considered to indicate excellent agreement.

All analyses were performed using Stata 16 (StataCorp. 2019. Stata Statistical Software: Release 16. College Station, TX: StataCorp LLC). A significance level of 0.05 was used throughout.

## Results

The demographic and body composition characteristics of the children recruited for the weight estimation studies used in this analysis are shown in Table 1.

**Table 1:** Demographic information for the children evaluated in each of the three studies. Abbreviations: IQR - interquartile range, BMI - body mass index.

	Study 1	Study 2	Study 3
Children evaluated	50/453	90	8
Datapoints for analysis	453	6720	264
Age (years) median (IQR)	3.8 (1.8 to 6.0)	7.3 (5.4 to 9.7)	10.0 (7.8 to 12.3)
Sex (male) n (%)	255 (56.3%)	46 (51.1%)	4 (50.0%)
BMI-for-age (centile) median (IQR)	50.0 (30.4 to 85.2)	54.9 (23.6 to 92.8)	67.5 (43.8 to 85)

Study 1

The inter-rater reliability analysis (in terms of accuracy of weight estimation) in 50 children who were measured by different observers showed a percentage agreement of 94% (47/50 cases) with a Cohen’s Kappa of 0.93 (0.81 - 1.0) (excellent agreement). The difference in weight estimation accuracy between major and minor data collectors in this study was not significant: major data collectors achieved accurate estimation within 10% of actual weight in 91% of cases and minor data collectors in 85% of cases (Fisher exact test, p=0.42).

Study 2

The inter-rater reliability analysis for habitus score assessment using intraclass correlation for the entire dataset of 6720 habitus score assessments was 0.73 (0.68 - 0.80) (good agreement). The results of the subgroup analyses are shown in Table 2. There were no significant differences between the rater groups or between the methods of assessing habitus.

**Table 2:** Results of subgroup analysis of intraclass correlation, by type of participant and method of habitus assessment. All data shown are the intraclass correlation coefficient with 95% confidence intervals.

	All participants	Advanced Life Support paramedics	Emergency Physicians	Paediatricians
All methods	0.73 (0.66- 0.8)	0.72 (0.65-0.8)	0.86 (0.82-0.9)	0.72 (0.64-0.79)
Gestalt habitus assessment	0.74 (0.64-0.83)	0.7 (0.59-0.81)	0.76 (0.67-0.86)	0.73 (0.63-0.83)
Reference-image assisted habitus assessment	0.72 (0.62-0.83)	0.74 (0.64-0.84)	0.73 (0.63-0.83)	0.71 (0.6-0.82)

Study 3

The inter-rater reliability analysis (in terms of achieving an accurate weight estimation) in this study with 33 raters and 8 children showed that the interrater reliability for the actual weight estimations had an ICC of 0.92 (0.83 - 0.98). There was an ICC of 0.88 (0.72 - 0.97) (good agreement) for an accuracy of estimations within 10% and 0.71 (0.30 - 0.93) (good agreement) for estimations within an accuracy of 20%.

## Discussion

There are three key determinants of a successful weight estimation system: accuracy, usability, and the ability to integrate with a comprehensive drug dosing guide [9]. The American Heart Association 2020 guidelines for paediatric advanced life support address the first two of these [11]:

“Several studies suggest that inclusion of body habitus or anthropometric measurements further refines and improves weight estimations using length-based measures. However, there is considerable variation in these methods, and the training required to use these measures may not be practical in every context.” 

With respect to accuracy, there is no doubt that the dual length- and habitus-based weight estimation systems (of which the PAWPER XL tape is one) have been established to be the most accurate methods available at present [6, 22, 23]. In terms of the practicality of the training required to use these systems, the comment in the guideline is misleading as all weight estimation systems require training for effective use. It has been proven that even the simplest of weight estimation methods, such as the Broselow tape, are susceptible to major errors when used by untrained or undertrained individuals [12]. The risk of these errors may also be increased in stressful resuscitation situations and when used in non-study conditions [16]. It is also worthwhile noting that the guidelines advocate for the use of length-based tapes with precalculated doses (of which the Broselow is the pre-eminent example) for weight estimation in emergencies. However, there has been very little data produced on the reliability of the tape. No data is available on the amount of training required for it to be used effectively. To express concern in the guideline about the potential training required for accurate weight estimation systems, while not acknowledging that training is still required for the methods that they advocate, is inconsistent and irrational.

Despite the fact that all weight estimation systems need to be practised to be used accurately, an analysis of inter-rater reliability in novice users may offer some insight into how much training is likely to be required for any individual system. In the secondary analysis in the current study on the PAWPER XL tape, the inter-rater reliability data from each of the three contributory studies ranged from good to excellent, irrespective of whether evaluating the reliability of the assessment of habitus score, the actual weight estimation, or the accuracy of the weight estimation. Since the methodology of the PAWPER XL system was designed to be resilient (it allows for different choices of habitus score to still result in an accurate weight estimation), it was encouraging that, when evaluating the reliability of achieving an accurate weight estimation, the tape performed consistently and with good reliably. These findings are thus reassuring that even with the minimal training provided in the studies, users can achieve accurate and consistent results.

No previous study has evaluated a weight estimation system in the same way that the current study has done. A previous study evaluating human factor errors with the use of the PAWPER XL tape, the Mercy method and the Broselow tape showed a variation between the accuracy achieved by different users, unrelated to training or prior experience [16]. Similarly, a study on the Mercy method, the Broselow tape and several older methods of weight estimation showed differences in the weight estimation accuracy achieved, with human factor errors contributing less to overall error than the limitations of the weight estimation methods themselves [15]. In these studies, the user errors related to the Broselow tape were of a similar magnitude to those of the more complex length- and habitus-based systems. It is clear that training is essential for any weight estimation system to function optimally (even the simplest methods). Future studies will need to clarify the type and frequency of training needed for users to perform optimally when using weight estimation systems. 

Limitations of this study

The accuracy of length measurements and mid-arm circumference were not directly assessed in Study 2. However, previous studies have suggested that the measurement of length and mid-arm circumference to have a high inter-rater reliability [24]. Study 1 and Study 2 were not conducted in real or simulated clinical scenarios, which could potentially falsely increase the accuracy of the estimations. Further large, prospective studies in different populations and settings, with a range of children of different ages and body types, and a range of raters with different levels of experience would be useful to provide additional supporting evidence on this matter.

## Conclusions

All three contributing studies showed a good to excellent inter-rater reliability in the use of the PAWPER XL tape systems. The analysis evaluated multiple aspects of the methodology, including the most important element, the final accuracy of weight estimation, and showed a similarly high level of inter-rater reliability in every step of the process. These findings suggest that the minimal training provided during the studies was sufficient to allow competent usage of the PAWPER XL tape by the participants. This data and previous studies suggest that the training required to permit effective usage of the PAWPER XL tape during emergencies could indeed be “practical in every context”.
